# Case Report: Atypical anti-KLHL11 antibody encephalitis: recurrent staring spells, serial negative MRI findings, and a tripartite diagnostic journey

**DOI:** 10.3389/fimmu.2026.1808939

**Published:** 2026-04-10

**Authors:** Yingyu Xie, Junling Chen, Weifan Xu, Yongdong Zhu, Houshi Zhou, Tongtong Cai

**Affiliations:** 1 Department of Neurology, Shantou Central Hospital, Shantou Clinical Medical College of Jinan University, Shantou, China; 2 Department of Electrophysiology, Shantou Central Hospital, Shantou Clinical Medical College of Jinan University, Shantou, China

**Keywords:** anti-KLHL11 antibody encephalitis, autoimmune encephalitis, case report, MRI-negative encephalitis, seizures

## Abstract

Anti-Kelch-like protein 11 (KLHL11) antibody encephalitis is a rare autoimmune neurological disorder, typically characterized by cerebellar syndrome and brainstem involvement, with magnetic resonance imaging (MRI) lesions predominantly localized to the brainstem and cerebellum consistent with rhombencephalitis. Epilepsy as the primary clinical presentation of this disease has been rarely reported. Herein, we describe a case of anti-KLHL11 encephalitis in which the patient presented with recurrent staring spells as the chief complaint, accompanied by transient mild cerebella signs. Brain imaging and routine cerebrospinal fluid (CSF) tests were unremarkable during the first two admissions; only generalized slow waves were detected on electroencephalogram (EEG) in the second admission. At the third admission, brain MRI revealed T2 hyperintensities in the left frontal and parietal lobes and CSF examination showed elevated protein levels; subsequent antibody testing confirmed anti-KLHL11-IgG positivity, thereby establishing a definitive diagnosis. The patient had negative results on tumor screening and exhibited a favorable response to therapy with corticosteroids, intravenous immunoglobulin and efgartigimod. This case highlights the diverse clinical manifestations and staged progression of anti-KLHL11 encephalitis, which warrants full recognition in clinical practice. For patients suspected of autoimmune encephalitis, dynamic re-examination of cranial imaging and CSF, as well as timely KLHL11-IgG testing, are critical for early diagnosis and prompt treatment, thereby improving prognosis. Additionally, this case expands the current understanding of anti-KLHL11 antibody encephalitis regarding its clinical presentations, imaging features, and therapeutic responsiveness.

## Introduction

Anti-KLHL11 antibody encephalitis is a subtype of autoimmune encephalitis. In 2019, Mandel-Brehm et al. first reported a young patient with seminoma presenting with ataxia, diplopia, and vertigo, and identified anti-KLHL11 antibodies using phage display technology, leading to the designation of anti-KLHL11 antibody encephalitis (1). Anti-KLHL11 encephalitis typically has an acute or subacute onset, mainly involving the brainstem and cerebellum. Its main clinical manifestations include ataxia, diplopia, vertigo, hearing loss, tinnitus, dysarthria, and other lower cranial nerve symptoms. Patients may also present with limbic encephalitis features such as seizures, psychiatric and behavioral disturbances, and cognitive decline (2). Anti-KLHL11 antibodies were initially recognized as a biomarker for specific malignancies, and subsequent studies confirmed that most patients with anti-KLHL11 antibody encephalitis are associated with tumors such as seminoma, leading to its classification as paraneoplastic KLHL11 encephalitis (2). On magnetic resonance imaging (MRI), abnormal T2-hyperintense signals in the cerebellum, brainstem, or diencephalon are commonly observed. As described above, given the predominant brainstem and cerebellar symptoms, this entity is also categorized within the spectrum of rhombencephalitis/brainstem encephalitis (3, 4).

Diagnosis of KLHL11 encephalitis presenting with epileptic symptoms is challenging due to the non-specific and insidious nature of its manifestations, especially when neuroimaging is negative, which may easily lead to misdiagnosis or underdiagnosis. Therefore, further accumulation of clinical experience is needed. Here, we report a patient with recurrent staring spells as the primary manifestation. Initial neuroimaging and routine cerebrospinal fluid (CSF) examinations were unremarkable. At the third admission, new abnormal lesions were detected in the left frontal and parietal lobes on brain MRI, and CSF analysis revealed elevated protein levels. Subsequent antibody testing confirmed positivity for KLHL11 antibodies, leading to a definitive diagnosis. Tumor screening was negative in this patient, and he showed a favorable response to treatment with corticosteroids, intravenous immunoglobulin, and efgartigimod.

## Case description

A 70-year-old male presented with recurrent episodes of staring spells and unresponsiveness. Each episode lasted 2–3 minutes, resolving spontaneously, and was followed by post-ictal headache and anterograde amnesia. Notably, no convulsions, trismus, orofacial frothing, or urinary/fecal incontinence were observed during these events. The patient’s personal history and past medical history were unremarkable. Regarding family history, the patient’s elder brother died of coronary heart disease many years ago; his younger brother has Parkinson’s disease; his mother has diabetes mellitus; and his three sisters are all healthy.

First Admission (October 2023)

**Figure 1:**
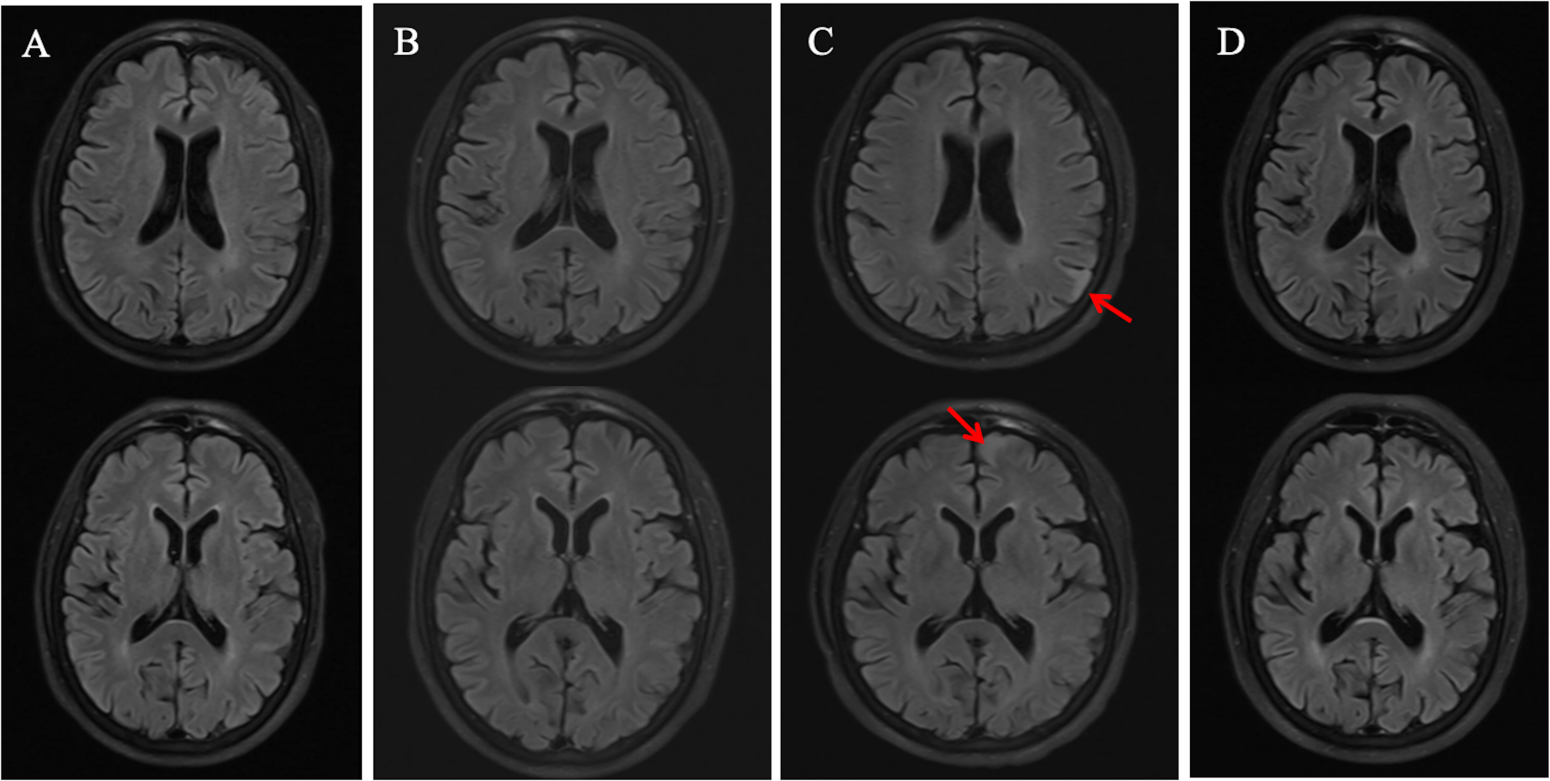
MRI changes at different admission. **(A)** First visit: No significant abnormalities were seen on the brain MRI. **(B)** Second visit: No significant abnormalities were seen on the brain MRI. **(C)** Third visit: T2-hyperintense signal (arrows) in the left frontal and parietal lobes. **(D)** Follow-up after treatment: The previously observed T-2 hyperintensities in the left frontal and parietal lobes had resolved.

**Figure 2:**
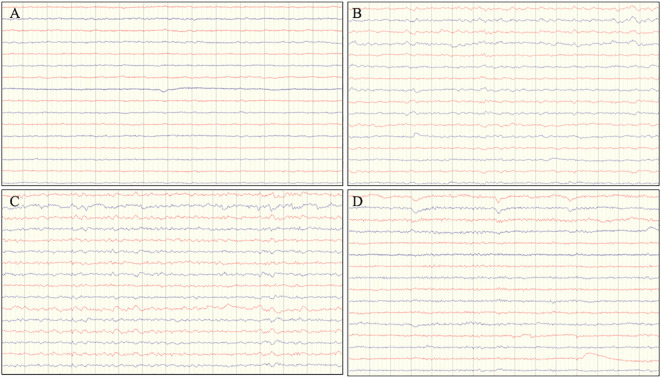
EEG changes at different admission. **(A)** First visit: No significant abnormalities. **(B)** Second visit: EEG demonstrated generalized slow-wave activity. **(C)** Third visit: EEG showed diffuse background slowing, with maximal involvement localized to the left anterior and posterior cerebral regions. **(D)** Follow-up after treatment: EEG demonstrated generalized slow waves with predominance in the frontopolar, frontal and temporal areas.

Contrast-enhanced brain MRI and electroencephalography (EEG) yielded unremarkable findings (**Figures 1A**, **2A**). Neuropsychological assessments revealed the following scores: Hamilton Anxiety Scale (HAMA)10, Hamilton Depression Scale (HAMD)5, Mini-Mental State Examination (MMSE)26, and Montreal Cognitive Assessment (MoCA) 19, suggestive of underlying anxiety. Although somatization disorder was initially hypothesized, the transient stereotyped nature of the episodes prompted clinical suspicion of epilepsy. Valproate sodium (0.4g,three times daily) was therefore initiated, take advantage of its dual efficacy in seizure control and mood stabilization.

Second Admission (November 2023)

One month after discharge with regular medication adherence, the patient experienced another staring spell while serving porridge, resulting in scalding of right hand. Neurological examination was unremarkable. Repeat contrast-enhanced brain MRI findings were consistent with the prior scan, whereas EEG demonstrated generalized slow-wave activity (**Figures 1B**, **2B**). Given the witnessed injury and EEG changes, imaging-negative autoimmune encephalitis (AE) was considered. CSF analysis showed normal intracranial pressure, protein, leukocytes, and glucose; CSF smear, culture, and cryptococcal antigen testing were all negative. The patient declined AE antibody testing due to financial limitations. Levetiracetam (1g, twice daily) was added, leading to complete seizure resolution, and close clinical follow-up was recommended.

Third Admission (January 2024)

The patient’s condition deteriorated with the onset of clustered seizures accompanied by behavioral abnormalities (repetitive light switching), with each episode prolonged to 15 minutes. Neurological examination revealed action tremor and impaired tandem gait; all other findings were unremarkable. Contrast-enhanced brain MRI revealed hyperintense signals on T2-weighted fluid-attenuated inversion recovery (T2/FLAIR) sequences in the left frontal and parietal lobes (**Figure 1C**). EEG showed diffuse background slowing, with maximal involvement localized to the left anterior and posterior cerebral regions (**Figure 2C**). Screening for connective tissue diseases, including antinuclear antibody spectrum, Hashimoto’s thyroiditis, and rheumatoid arthritis, yielded negative results. Lumbar puncture showed elevated CSF protein levels with normal leukocyte count, glucose concentration, and chloride; bacterial/fungal cultures and metagenomic capture-based pathogen nucleic acid high-throughput sequencing were negative. AE was thus highly suspected. We once again recommended comprehensive testing for autoimmune encephalitis–related antibodies. New cranial imaging findings strongly supported the diagnosis of AE and improved the family’s acceptance. Although initially hesitant for financial reasons, the family eventually consented after the patient’s seizures proved refractory to escalated antiseizure medications, which further increased caregiver burden and posed risks of burns, falls, and electric shock. They completed the testing after securing financial assistance from other relatives. Given the non-specific seizure manifestations, imaging findings, and newly identified subtle cerebellar signs, AE antibody testing (KLHL-11, NMDAR, AMPAR1, AMPAR2, LGI1, CASPR-2, GABABR, DPPX, IgLON5, GAD65, GlyR1, D2R, mGlu5, mGlu1, Neurexin3ɑ,GABAARα1, GABAARβ3) and demyelinating antibodies (AQP4, MOG, GFAP) were performed, which confirmed positive anti-KLHL11 antibodies (CSF titer 1:10; serum titer 1:320). Oligoclonal band testing and IgG index were not performed. Considering the strong association between anti-KLHL11 antibodies and malignancies (particularly genitourinary neoplasms), tumor marker screening, scrotal ultrasonography, and whole-body ^18^F-fluorodeoxyglucose positron emission (^18^F-FDG PET-CT) were conducted, all of which showed no evidence of malignancy.

The patient received intravenous methylprednisolone (1000 mg/day for 3 days, followed by tapering doses of 500 mg/day, 250 mg/day, and 120 mg/day for 3 days each), intravenous immunoglobulin (IVIG,0.4g/kg/d for 5 days), and was maintained on prednisone (tapered to 10 mg/day), valproate extended-release (0.5g, twice daily), and azathioprine (100 mg/day).

A 3-month follow-up contrast-enhanced brain MRI showed near-complete resolution of the previously noted lesions (**Figure 1D**). EEG demonstrated generalized slow waves with predominance in the frontopolar, frontal and temporal areas(**Figure 2D**). Repeat anti-KLHL11 antibody testing in both serum and CSF turned negative, and the patient remained seizure-free for 1 year.

Fourth Admission (September 2025)

**Figure 3:**
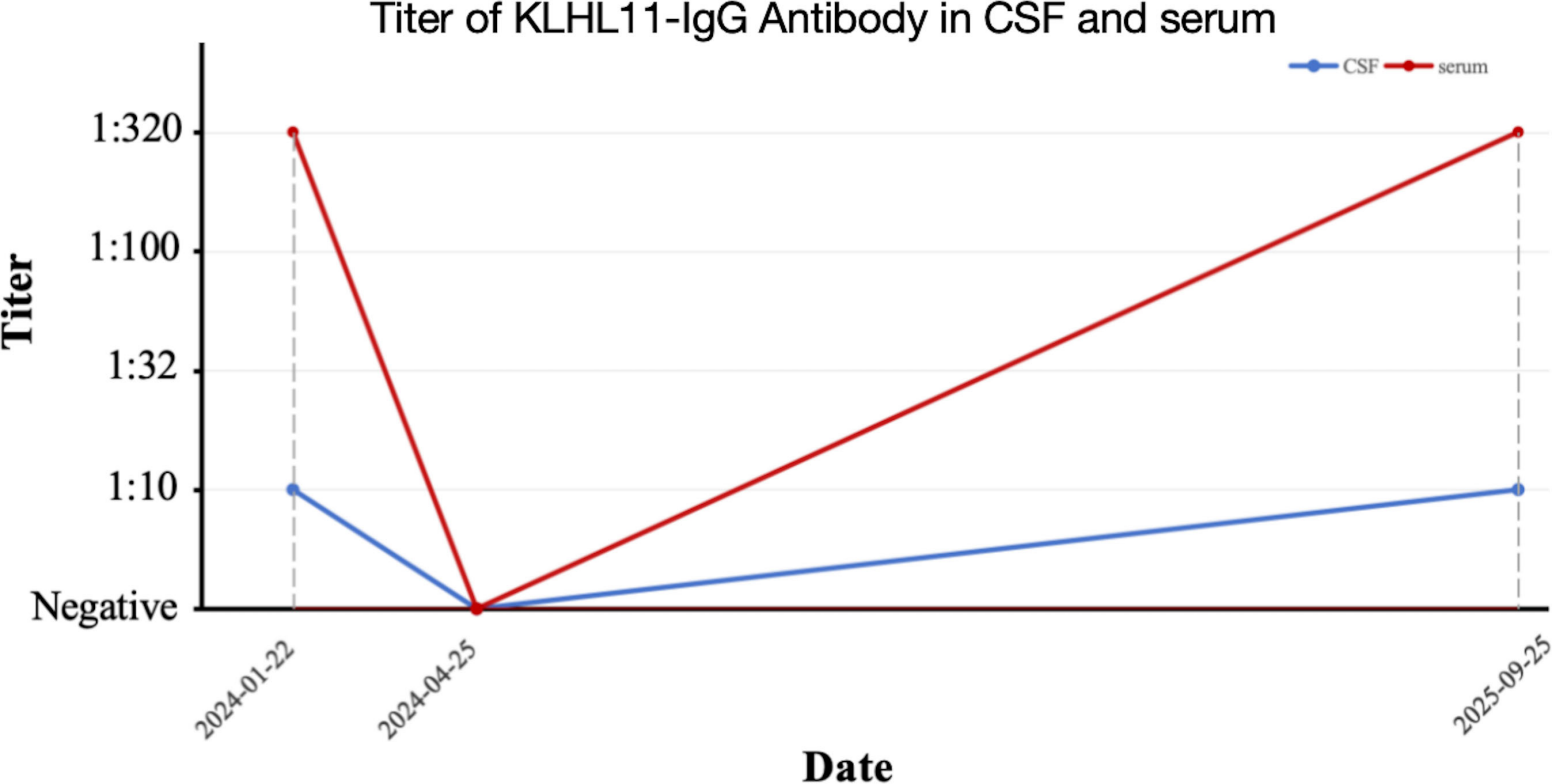
Longitudinal changes in cerebrospinal fluid and serum KLHL11-IgG titers during the clinical course.

Despite persistent adherence to the maintenance medication regimen, the patient developed recurrent refractory seizures that were unresponsive to the addition of levetiracetam. Contrast-enhanced brain MRI findings were stable, while repeat anti-KLHL11 antibody testing was positive again (CSF titer 1:10; serum titer 1:320), confirming a relapse of anti-KLHL11 antibody encephalitis. Longitudinal changes in cerebrospinal fluid and serum KLHL11-IgG titers during the clinical course were presented in **Figure 3**. Re-administration of methylprednisolone pulse therapy reduced seizure frequency but failed to achieve complete seizure control. The patient had experienced mild weight gain (∼3 kg) during initial methylprednisolone pulse therapy, followed by continuous weight gain (total 15 kg from baseline) and hyperglycemia during subsequent oral prednisone. Thus, the patient requested minimization of steroid exposure. Efgartigimod (1200 mg IV weekly for 2 weeks) was added, resulting in complete cessation of seizures. The patient continued maintenance therapy with low-dose prednisone combined with azathioprine, with a prospective plan to evaluate efgartigimod as a long-term steroid-sparing agent. Repeat malignancy screening, including contrast-enhanced thoracoabdominal computed tomography (CT) and genitourinary ultrasonography, yielded.

## Discussion

Several clinical features rendered the diagnosis of our patient particularly challenging. First, although anti-KLHL11 antibody encephalitis can manifest with limbic encephalitis symptoms, such presentations are not typical. Epileptic seizures, as the initial clinical manifestation in this case, lack specificity. Transient and subtle cerebellar signs emerged only during disease progression, which should alert clinicians to consider cerebellum-involving encephalitis subtypes. However, such mild signs are easily overlooked, leading to diagnostic delays or inaccuracies. Second, the patient’s imaging findings were non-specific. Previous reports indicate that anti-KLHL11 antibody encephalitis typically exhibits characteristic neuroimaging abnormalities, including brainstem and cerebellar atrophy, as well as T2/FLAIR hyperintensities in multiple brain regions such as the brainstem, cerebellum, diencephalon, and temporal lobe (4). In contrast, our patient showed no specific abnormalities on multiple early imaging examinations. Notably, the newly developed lesions in the left frontal and parietal lobes during disease progression have not been previously reported in the literature. Following subsequent immunotherapy, the anti-KLHL11 antibodies turned negative, and the frontal and parietal lesions achieved near-complete resolution. These findings suggest that the neuroimaging manifestations of anti-KLHL11 antibody encephalitis are heterogeneous, which may be correlated with disease stages and the intensity of immune responses. Furthermore, the patient’s initial CSF routine tests were negative, while the second CSF examination showed mildly elevated protein levels. In contrast, previously reported cases commonly present with elevated CSF protein levels (range: 23–200 mg/dL, median: 65 mg/dL), pleocytosis (range: 0–86/μL, median: 10/μL, predominantly lymphocytes), positive oligoclonal bands, and elevated IgG index, which are distinctly different from the findings in our patient (2).

A strong association between anti-KLHL11 antibody encephalitis and underlying malignancies has been well established: among 133 reported cases, 108 patients (81.2%) had concurrent tumors, with testicular seminoma being the most prevalent, followed by ovarian teratoma. Other associated tumor types include ovarian cancer, breast cancer, thymic germ cell tumors, small cell lung cancer, lung adenocarcinoma, and leukemia (5). Therefore, routine tumor screening is recommended for patients with anti-KLHL11 antibody encephalitis, and ^18^F-FDG PET-CT is helpful for identifying potential underlying malignancies. In our case, no malignant lesions were detected on PET/CT and scrotal ultrasonography, and no tumor-related manifestations emerged during nearly 2 years of follow-up. This may account for the favorable therapeutic response observed in the patient, though long-term follow-up and surveillance remain necessary.

Like most autoimmune encephalitides, anti-KLHL11 antibody encephalitis usually presents with an acute or subacute onset. The most common clinical manifestation is rhombencephalitis (involving the brainstem and/or cerebellum), followed by cochleovestibular disorders and limbic encephalitis. Among 133 reported cases, common clinical manifestations included ataxia (61.6%), dizziness (35.3%), diplopia (26.3%), and hearing loss (23.3%). Seizures (9.7%) and cognitive impairment (4.5%) were less common and mostly occurred as accompanying features (5). The present patient predominantly presented with recurrent seizures without typical features of rhombencephalitis, highlighting the clinical heterogeneity of anti-KLHL11 antibody encephalitis and posing certain diagnostic challenges. The MATCH score — M (male, 1 point), A (ataxia or other cerebellar signs, 1 point), T/C (testicular tumor, 2 points; other tumor types, 1 point), H (hearing impairment, 1 point) — is designed to identify patients for whom KLHL11 antibody testing is indicated. A score ≥ 4 indicates a higher probability of anti-KLHL11 antibody encephalitis (sensitivity = 78%, specificity = 99%) (6). However, this score overemphasizes brainstem and cerebellar symptoms as well as the presence of an associated tumor, resulting in relatively low sensitivity for atypical presentations. Our patient presented with seizures as the predominant manifestation, exhibited only transient and equivocal cerebellar signs (action tremor) during the clinical course, and had no associated tumor, yielding a maximum MATCH score of 2. This indicates that the MATCH score has low sensitivity for detecting non-classic anti-KLHL11 antibody encephalitis. Therefore, when diagnosing KLHL11 encephalitis, it is critical to recognize that the disorder may present with seizures as the sole major clinical manifestation, in the absence of rhombencephalitis-related features. Importantly, anti-KLHL11 encephalitis can manifest as an isolated/primary autoimmune encephalitis of the nervous system and is not invariably associated with an underlying malignancy.

To date, there is no standardized treatment protocol for anti-KLHL11 antibody encephalitis. Current management strategies are extrapolated from those for other paraneoplastic encephalitis, including immunotherapy and management of potential underlying malignancies. In this case, the patient received not only first-line immunotherapies—including corticosteroids and IVIG—during the acute phase but also efgartigimod, an additional immunotherapeutic agent. Efgartigimod is a neonatal Fc receptor (FcRn) antagonist that exerts therapeutic effects in autoimmune diseases by reducing circulating IgG antibody levels, thereby lowering the concentration of pathogenic IgG autoantibodies (7). Moreover, efgartigimod inhibits FcRn-regulated presentation of antigen-IgG immune complexes (ICs) in antigen-presenting cells (e.g., dendritic cells and macrophages), which in turn suppresses MHC class II-mediated CD4+ T cell activation and MHC class I-mediated CD8+ T cell cytotoxicity. Concurrently, IgG-IC-mediated complement activation is attenuated. On the other hand, efgartigimod impedes the release of proinflammatory cytokines (e.g., IL-6, TNF-α, and IFN-γ), which are triggered by IgG binding to Fcγ receptors (FcγRs) on macrophages and dendritic cells (8, 9). Previous reports have documented symptomatic improvement in anti-KLHL11 antibody encephalitis patients treated with efgartigimod (4, 9). In our case, the patient’s clinical manifestations improved significantly following efgartigimod administration, suggesting its potential therapeutic value, though further long-term follow-up is required to determine the sustained treatment response.KLHL11 antibody encephalitis was previously thought to be mediated solely by cellular immunity; however, the responsiveness to efgartigimod suggests a potential involvement of antibody-mediated immunity.

In conclusion, this case expands the spectrum of clinical and neuroimaging phenotypes of anti-KLHL11 antibody encephalitis. It highlights that clinicians should recognize the atypical manifestations of this disease. Timely completion of antibody testing, repeated neuroimaging evaluations, and early initiation of immunotherapy are crucial for improving diagnostic accuracy and optimizing patient prognosis. The successful use of efgartigimod in this case also suggests that antibody-mediated immunity may serve as a potential therapeutic target for anti-KLHL11 antibody encephalitis.

## Data Availability

The original contributions presented in the study are included in the article/supplementary material. Further inquiries can be directed to the corresponding authors.
